# Genomic Diversity and Evolution of *Mycobacterium ulcerans* Revealed by Next-Generation Sequencing

**DOI:** 10.1371/journal.ppat.1000580

**Published:** 2009-09-11

**Authors:** Weihong Qi, Michael Käser, Katharina Röltgen, Dorothy Yeboah-Manu, Gerd Pluschke

**Affiliations:** 1 Department of Medical Parasitology and Infection Biology, Swiss Tropical Institute, Basel, Switzerland; 2 Department of Bacteriology, Noguchi Memorial Institute for Medical Research, University of Ghana, Legon, Ghana; The Pennsylvania State University, United States of America

## Abstract

*Mycobacterium ulcerans* is the causative agent of Buruli ulcer, the third most common mycobacterial disease after tuberculosis and leprosy. It is an emerging infectious disease that afflicts mainly children and youths in West Africa. Little is known about the evolution and transmission mode of *M. ulcerans*, partially due to the lack of known genetic polymorphisms among isolates, limiting the application of genetic epidemiology. To systematically profile single nucleotide polymorphisms (SNPs), we sequenced the genomes of three *M. ulcerans* strains using 454 and Solexa technologies. Comparison with the reference genome of the Ghanaian classical lineage isolate Agy99 revealed 26,564 SNPs in a Japanese strain representing the ancestral lineage. Only 173 SNPs were found when comparing Agy99 with two other Ghanaian isolates, which belong to the two other types previously distinguished in Ghana by variable number tandem repeat typing. We further analyzed a collection of Ghanaian strains using the SNPs discovered. With 68 SNP loci, we were able to differentiate 54 strains into 13 distinct SNP haplotypes. The average SNP nucleotide diversity was low (average 0.06–0.09 across 68 SNP loci), and 96% of the SNP locus pairs were in complete linkage disequilibrium. We estimated that the divergence of the *M. ulcerans* Ghanaian clade from the Japanese strain occurred 394 to 529 thousand years ago. The Ghanaian subtypes diverged about 1000 to 3000 years ago, or even much more recently, because we found evidence that they evolved significantly faster than average. Our results offer significant insight into the evolution of *M. ulcerans* and provide a comprehensive report on genetic diversity within a highly clonal *M. ulcerans* population from a Buruli ulcer endemic region, which can facilitate further epidemiological studies of this pathogen through the development of high-resolution tools.

## Author Summary

*Mycobacterium ulcerans* is the causative agent of Buruli ulcer (BU), a necrotizing skin disease and the third most common mycobacterial disease after tuberculosis and leprosy. It is an emerging infectious disease that afflicts mainly children and youths in West Africa. The disease is also found in tropical and subtropical regions of Asia, the Western Pacific, and Latin America. Limited knowledge of this neglected tropical disease is partially due to the lack of known genetic polymorphisms among isolates, which hinder the study of transmission, epidemiology, and evolution of *M. ulcerans*. Our aim is to systematically profile genetic diversity among *M. ulcerans* isolates by sequencing and comparing the genomes of selected strains. We identified single nucleotide polymorphisms (SNPs) within a highly clonal *M. ulcerans* population from a Buruli ulcer endemic region. Based on the SNPs discovered, we developed SNP typing assays and were able to differentiate a collection of *M. ulcerans* isolates from this Buruli ulcer endemic region into 13 SNP haplotypes. Our results lay the ground for developing a highly discriminatory and cost-effective tool to study *M. ulcerans* evolution and epidemiology at a population level.

## Introduction

*Mycobacterium ulcerans* causes Buruli ulcer (BU), a necrotizing skin disease and the third most common mycobacterial disease, after tuberculosis and leprosy [1]. In the past decade the incidence of BU has increased dramatically in West African countries, but the disease is also found in tropical and subtropical regions of Asia, the Western Pacific, and Latin America [2]. Due to the focal distribution of the disease and the fact that it affects mainly poor rural communities, BU belongs to the neglected tropical diseases. Limited knowledge about the disease is partially caused by the lack of molecular fine-typing methods, which hinder the study of transmission, epidemiology, and evolution of the clonal pathogen.

Genetic analyses suggested that *M. ulcerans* diverged from the fish pathogen *M. marinum* between 470,000 and 1,200,000 years ago by acquiring the virulence plasmid pMUM001 [3],[4]. Whole genome comparison of *M. marinum* strain M and *M. ulcerans* strain Agy99 revealed that the two strains share >98% nucleotide sequence identity, although extensive DNA insertions and deletions have been observed [4]. Our recent comparative genomic hybridization study found extensive large sequence polymorphisms (LSPs) among *M. ulcerans* clinical isolates of diverse geographic origins. Two distinct *M. ulcerans* lineages have been defined: the ancestral lineage of strains from Asia, South America and Mexico, which are genetically closer to the progenitor *M. marinum*, and the classical lineage of strains from Africa, Australia and South East Asia [5],[6].

Although continental types of *M. ulcerans* strains have been well established, differentiation between isolates within a geographic region, such as strains from African countries, has remained a challenge [7],[8]. Different genotyping methods have been applied to *M. ulcerans*, including IS*2426* polymerase chain reaction (PCR) [9],[10], amplified fragment length polymorphism (AFLP) [11], IS*2404* restriction fragment length polymorphism (RFLP) [12],[13], multi-locus sequence typing (MLST) [3],[14],[15], variable-number tandem repeat (VNTR) typing [7],[8],[16],[17] and IS*2404*-Mtb2 PCR [18]. Among these, AFLP [11] and recently established VNTR typing methods [7],[8] were the only techniques that have provided some resolution among clinical strains of *M. ulcerans* from Africa, confirming that genotypic diversity attributable to insertions, deletions, and duplications of variable DNA fragments exists among African strains. However, protein coding genes in *M. ulcerans* African populations harbor extremely low levels of polymorphisms. No single nucleotide polymorphisms (SNPs) were detected in a multi locus sequence typing of a few chromosomal and plasmid genes [3],[14],[15].

To systematically and comprehensively study the genetic diversity and the evolution of *M. ulcerans* strains, a genome wide profiling is needed. The complete genome sequence of *M. ulcerans* Agy99 consists of a circular chromosome of 5632 Kb and a plasmid pMUM001 of 174 Kb [4], which can be used as the reference for comparative genome analysis. The ongoing revolution in massively parallel sequencing technology [19],[20], such as the availability of Roche 454 Life Sciences Genome Sequencer FLX [21], Applied Biosystems SOLiD System, and Illumina Solexa Genome Analyzer [22], has made it possible to sequence large numbers of bacterial strains within days [23]. Next generation sequencing platforms have been used for genome wide profiling of novel genetic variations in many different organisms, including viruses [24], bacteria [25],[26],[27], plants [28],[29], worms [30] and humans [31],[32]. Here we report the sequencing of the genomes of three selected *M. ulcerans* strains using pyrosequencing (Roche 454 Life Science) and Solexa (Illumina) sequencing by synthesis technologies. Based on these sequences we identified SNPs, which we used to estimate evolutionary times for the emergence of *M. ulcerans*. We also developed SNP typing assays as high-resolution genotyping methods for *M. ulcerans*. Genetic fingerprinting of bacterial isolates will be a valuable tool for distinguishing relapses from new infections, tracing infection chains, and identifying environmental reservoirs. Molecular epidemiological analyses based on SNP typing may finally contribute to better disease control by identifying preventable risks for infection.

## Results

### Selection of *M. ulcerans* strains for genome sequencing

With the aim to comprehensively investigate genome diversity of *M. ulcerans* strains from an individual geographical region, we selected two Ghanaian patient isolates from different residential districts and sequenced their genomes with 454 and Solexa technologies, respectively (Table 1). These two strains were isolated from the same African country as the fully sequenced reference strain Agy99 [4]. Whereas Agy99 was isolated in 1999 from a BU patient, the two selected Ghanaian patient isolates NM20/02 and NM31/04 were isolated after an apparent process of replacement of VNTR types in Ghana [7] in the years 2002 and 2004, respectively. Agy99, NM31/04 and NM20/02 represent the three VNTR types (Table 1) previously identified in Ghana [7]. While the Ghanaian strains belong to the classical lineage, we also included a Japanese patient isolate, ITM Japan8756 (denoted as Jp8756 from here on in the paper), as a representative of the ancestral lineage in our analysis. Its genome was sequenced with the Solexa Genome Analyzer. Selected genomic regions of this strain were also sequenced with a NimbleGen comparative genome sequencing (CGS) microarray and the results were compared.

**Table 1:** *Mycobacterium ulcerans* strains sequenced in this study.

Strain	Year of isolation	Place of origin	MIRU 1 allele^2^	STI allele^2^
NM20/02	2002	Ga District, Greater Accra region, Ghana	B	BD
NM31/04	2004	Amansie West District, Ashanti region, Ghana	BAA	C
Jp8756	1980	Japan	nd^3^	CF
Agy99^1^	1999	Ga district, Greater Accra region, Ghana	BAA	BD

1 Reference strain

2 Hilty et al., 2006 [7]

3 Not determined

### Single nucleotide polymorphisms in *M. ulcerans* strains

We sequenced three *M. ulcerans* strains with single end reads generated by two different next–generation sequencing platforms. For NM20/02, we obtained 424,494 GS FLX reads (Roche 454) with an average length of 213 bases. For NM31/04 and Jp8756, we obtained 2.5 and 2.7 million 35-bp Solexa reads, respectively (Table 2). To identify SNPs, we mapped the reads to the reference genome, including both the Agy99 chromosome and the plasmid pMUM001.

**Table 2:** Summary of next generation sequencing results.

Strain		NM20/02	NM31/04	Jp8756
Sequencing method		Roche 454 GS FLX	Illumina Solexa GA	Illumina Solexa GA
Total no. Reads		424,494	2,538,429	2,651,276
Averaged read length (nt)		213	35	35
Total sequences (nt)		90,299,836	88,845,015	92,794,660
Map to Agy99 chromosome	Total no. reads mapped (%)	382,116 (90.01)	2,343,269 (92.31)	2,279,741 (85.99)
	% genome mapped	93.72	99.99	94.47
	Average depth of mapped regions	14.5	14.1	13.6
Map to Agy99 plasmid pMUM001	Total no. reads mapped (%)	5,269 (1.24)	326,541 (12.86)	89,325 (3.37)
	% genome mapped	32.56	100	20.35
	Average depth of mapped regions	19.8	63.4	17.2

We used the 454 software gsMapper for GS FLX reads and MAQ [33] for Solexa reads. The MAQ places Solexa reads mapped to multiple locations randomly, while gsMapper excludes reads mapped to repeated regions, such as insertion sequences (IS)*2404* and IS*2606*, which are present in high copy numbers in the *M. ulcerans* genome [4]. Therefore, the Agy99 chromosome was better covered by Solexa reads than by GS FLX reads. 94% of the Agy99 chromosome was mapped with NM20/02 GS FLX reads, 99.99% with NM31/04 Solexa reads, 94.47% with Jp8756 Solexa reads. The average depth was 14 to 15 fold. We identified 135 chromosomal SNPs in NM20/02, 83 SNPs in NM31/04, and 26,564 SNPs in Jp8756 (Table S1).

The coverage for pMUM001 varied a lot from strain to strain. 33% of the pMUM001 was mapped with NM20/02 reads, 100% with NM31/04 reads, and 20% with Jp8756 reads. The average depth ranged from 63 for NM31/04 reads to 17 for Jp8756 reads (Table 2). Because the low coverage of the plasmid in NM20/02 could be an artifact from the gsMapper, which excluded reads mapped to non-unique regions, we mapped NM20/02 reads to pMUM001 using another software, MOSAIK (http://bioinformatics.bc.edu/marthlab/Mosaik), which allowed us to compare mapping results with non-uniquely mapped reads included or excluded. When all mapped reads were assembled regardless of their uniqueness, the full length of pMUM001 was well covered (Figure S1 A), suggesting the presence of a pMUM001-like plasmid in NM20/02. When only uniquely mapped reads were recorded, pMUM001 was partially covered (Figure S1 B). Reads mapped to regions such as those encoding Type I modular polyketide synthase genes, transposase genes, IS elements (IS*2606* and IS*2404*) were excluded due to their non-uniqueness. Although mapping analysis using MOSAIK confirmed that the lack of pMUM001 coverage in NM20/02 was a data analysis artifact, the lack of pMUM001 in Jp8756 was confirmed. MAQ didn't exclude non-uniquely mapped reads and was able to map the full length of pMUM001 with NM31/04 reads, which were analyzed exactly the same way as Jp8756 reads. Mapping using MOSAIK not only confirmed the lack of pMUM001 coverage by Jp8756 reads (Figure S1 E), but also revealed that the depth of pMUM001 regions covered by uniquely mapped Jp8756 reads were very low, which ranged from one read to four reads (Figure S1 F), while the depth of pMUM001 regions covered by uniquely mapped reads ranged from 10 to 50 in NM20/02 and NM31/04 (Figure S1 B and D). Most likely the under-representation of plasmid DNA in the total DNA sample is due to either complete or partial loss of plasmid sequences, which is frequently found in *M. ulcerans* strains that have been cultured over extended periods of time. Previous plasmid sequences analyses have found that plasmids in Japanese and African *M. ulcerans* strains are highly conserved in size and sequence [15],[34]. However, a recent study found different strains of *M. ulcerans* were capable of producing structurally distinct mycolactones, which could be due to presence of sequence variations in pMUM001[35]. Our study suggests that the plasmids from all three African strains are highly similar. We only found one intergenic SNP and one synonymous SNP shared by NM31/04 and NM20/02 plasmids. The two non- synonymous SNPs found in the NM20/02 plasmid were within *IS*2606 genes. In Jp8756 we identified one intergenic SNPs in the plasmid regions with sufficient coverage (Table S1). To elucidate how the sequence variations affect mycolacton production, future experiments in the lab will be needed to enrich and analyze the plasmids in each test strain, together with characterization of mycolacton production.

Before next generation sequencing technologies became widely available, we have sequenced Jp8756 using comparative genome sequencing microarrays covering selected regions of the Agy99 chromosome and pMUM001. A total of 1,618 SNPs were identified in the selected 1.2 Mb chromosomal protein coding regions. 1,389 (86%) of these SNPs were confirmed by Solexa sequencing (Table S2). On both mutation mapping arrays and re-sequencing arrays, probes targeting pMUM001 showed very low signals (Figure S2), as compared to probes targeting the Agy99 chromosome, which also confirmed the lack of plasmid DNA in the Jp8756 DNA sample.

**Figure 1:**
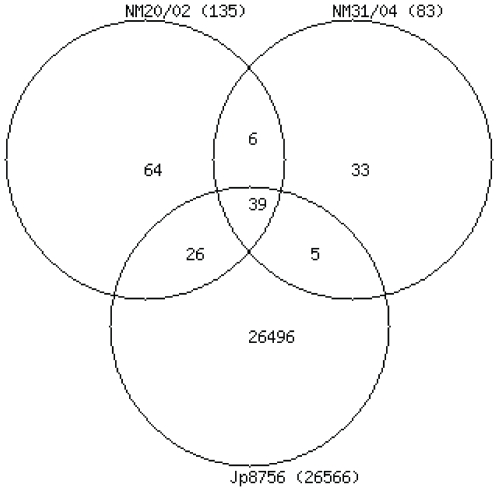
Venn diagram of single nucleotide polymorphisms in *M. ulcerans* strains.

In total 26,669 SNPs were identified by comparing the Jp8756 to the Agy99 chromosome; 18,510 in 3,597 protein coding genes, and 8,159 in 1,768 different intergenic regions (Table S1). 99.35% of the SNPs were found only in the Japanese strain (Figure 1). In comparison to Agy99, the average number of SNPs ranged from 1 per 210 bp in Jp8756 to 1 per 68 Kb in NM31/04. While the Japanese strain Jp8756 and the Ghanaian strain Agy99 share 99.53% nucleotide sequence identity, the average percentage of polymorphic nucleotide sites between Ghanaian strains is only 0.0015% (NM31/04 vs. Agy99) and 0.0024 (NM20/02 vs. Agy99), respectively. These results are consistent with previous findings that strains of the ancestral lineage are genetically distant to the classical lineage strains [6]. There are 103 SNPs specific to the Ghanaian strains with an average of 0.0018% of polymorphic nucleotide site differences from the Agy99 genome (Figure 1).

**Figure 2:**
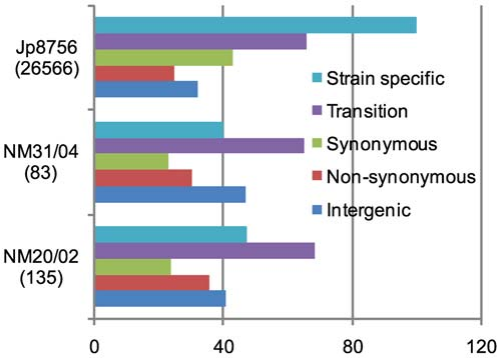
Summary of single nucleotide polymorphisms in *M. ulcerans* strains. The number in parentheses represents the total number of SNPs in each sequenced strain. The color bars show the distributions of different SNP categories in each strain.

We classified identified SNPs into five different categories: strain specific SNPs, transversions, synonymous SNPs (sSNPs), non-synonymous SNPs (non-sSNPs), and intergenic SNPs. Figure 2 shows the total numbers of SNPs and the percentage of different categories of SNPs found in each strain. Overall, only 34 SNPs were found parsimoniously informative (PI), common to at least two strains or “non-strain-specific.” The numbers of “strain-specific” SNPs varied from 33 (40% of 83 SNPs) in NM31/04 to 26,496 (99.7% of 26564 SNPs) in Jp8756. In all three strains the percentage of intergenic SNPs was around 40%, indicating a similar distribution of point mutations in coding and non-coding regions. Interestingly, the majority of coding region SNPs (63%) found in Jp8756 was synonymous, while in the two Ghanaian strains the majority was non-synonymous. About 100 non-synonymous SNPs found in strain Jp8756, but none of the non-synonymous SNPs found in the two Ghanaian strains caused premature stop codons. While the accumulation of pseudogenes seems to play an important role in both the divergence of *M. ulcerans* from *M. marinum* and the emergence of *M. ulcerans* continental types [4], our observation suggests that there is no further formation of pseudogenes within the studied Ghanaian strains. The percentage of SNP transition ranged between 65% and 68%, suggesting a substitution bias in favor of nucleotide substitution within the purine or pyrimidine group.

We did not try to identify insertions or deletions (indels) of any size. First of all, single-base/small indels were difficult to identify based on 454 data due to higher indel error rate observed in pyrosequencing [36]. Secondly, identification of single-base/small indels using short reads, such as solexa or SOLiD data, requires paired-end data [33], which were not provided in our study. Without paired-end data, discovery of single-base/small indels using short reads requires gapped alignments of millions of short reads, which is computational challenged. Most currently available short read mapping tools supports only non-gapped alignments. Gapped alignment tools such as BLAST is not feasible for mapping short reads in a reasonable time scale and not guaranteed to give the right answer [37]. Last but not least, identification of large indels with high confidence also requires paired-end data. This is because missing regions can be explained by other factors, such as insufficient sequencing coverage, differences in mapping algorithms when dealing with reads mapped to repeated regions, and so on.

### Genes potentially under selections

**Figure 3:**
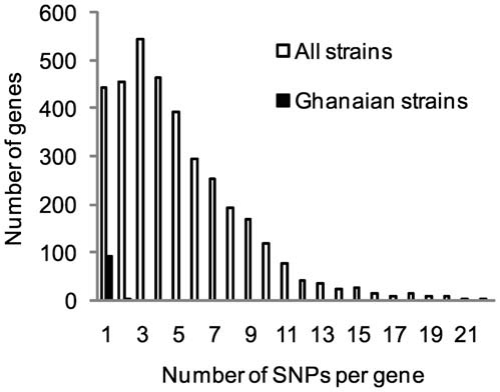
Distribution of number of SNPs per gene.

Among the 94 protein coding genes containing SNPs found in the Ghanaian strains, most genes harbored only one SNP (Figure 3). Only three genes contained two SNPs: MUL_2118 (hypothetical protein), MUL_3524 (diphosphomevalonate decarboxylase) and MUL_3716 (nucleoside diphosphate kinase). When considering all strains, 3,597 genes harbored SNPs, most of which contained one to five SNPs (Figure 3), but much higher numbers of SNPs per gene were also observed. For example, the peptide synthetase Nrp gene MUL_2638 contained 59 SNPs, the Pks12 gene MUL_2266 harbored 55 SNPs, and the fatty acid synthase Fas gene MUL_3818 had 30 SNPs. However, all three genes were over 9 Kb in length, which may account for the high number of SNPs. We did find 70 genes with a SNP density higher than 1 per 80 bp, which was one standard deviation higher than the average SNP density (Table 3). More than 50% of these consisted of genes encoding hypothetical proteins. Others included genes associated with antigenic proteins (i.e. *esxE*, *esxF*, *mpt64*), lipoproteins (i.e. *dsbF*, *lppN*, *lpqV*), PE/PE-PGRS family proteins (MUL_4359, MUL_0355), membrane proteins (*mmr*) and transcription regulators (MUL_2645, MUL_0993). The high number of SNPs may represent evidence for selection pressure on these genes. We thus calculated values of synonymous differences per synonymous site (*p_S_*) and nonsynonymous differences per nonsynonymous site (*p_N_*) across all SNP harboring loci. The average *p_N_*−*p_S_* value was −0.00276±0.0073 (P<0.001, *p_S_* = 0.0073±0.0063, *p_N_* = 0.0046±0.0033), suggesting that on average the frequency of synonymous mutation was significantly higher than the non-synonymous mutation frequency; i.e. there was no evidence for diversifying selection. Among the 70 genes found earlier with high SNP density, five genes (*esxE*, *mmr*, MUL_0355, as well as the hypothetical protein genes MUL_0161 and MUL_2106) showed significantly high *p_N_*−*p_S_* values (above the mean + 3× standard deviations) and might be under diversifying selection, while six genes (*glbO*, *mcmA2b*, MUL_1435, MUL_2645, MUL_4312 and MUL_4846) showed significantly low *p_N_*−*p_S_* values (less than the mean−3× standard deviations), which might be under negative selection (Table 3). Meanwhile, we found 32 genes with average SNP density but significant low *p_N_*−*p_S_* values, including genes related to transcription and translation (*whiB4*, *trxC*, *trpG*, MUL_0058, MUL_2937, MUL_4776) and another ESAT-6 family protein gene, *esxR* (Table 3).

**Table 3:** Genes potentially under selection ordered by SNP density.

Locus_tag	Locus	Product	COG	SNP density (bp per SNP)	Selection^1^
MUL_3769	-	hypothetical protein	-	39	
MUL_4312	-	hypothetical protein	-	41	−
MUL_4235	-	hypothetical protein	-	45	
MUL_5054	*esxE*	ESAT-6 like protein EsxE	COG1314U	46	+
MUL_3425	*mmr*	multidrug-transport integral membrane protein Mmr	-	46	+
MUL_1135	-	hypothetical protein	COG1902C	46	
MUL_5072	*gid*	glucose-inhibited division protein B Gid	-	47	
MUL_4359	-	PE family protein	COG0357M	48	
MUL_3746	*glbO*	globin (oxygen-binding protein) GlbO	-	48	−
MUL_4906	-	hypothetical protein	-	50	
MUL_0630	-	hypothetical protein	COG0500QR	51	
MUL_5017	-	hypothetical protein	-	52	
MUL_4764	-	hypothetical protein	COG0500QR	54	
MUL_2201	-	hypothetical protein	-	55	
MUL_0760	-	hypothetical protein	-	56	
MUL_3596	-	hypothetical protein	-	56	
MUL_1662	*gloA*	lactoylglutathione lyase, GloA	-	56	
MUL_2106	-	hypothetical protein	COG0315H	57	+
MUL_4509	-	hypothetical protein	-	57	
MUL_4133	*dsbF*	lipoprotein DsbF	-	58	
MUL_0355	-	PE-PGRS family protein family protein	COG2346R	60	+
MUL_3885	*echA4_2*	enoyl-CoA hydratase, EchA4_2	COG1773C	60	
MUL_5108	-	transposase	COG1119P	60	
MUL_0384	-	hypothetical protein	-	61	
MUL_5010	-	phosphoglycerate mutase	COG0406G	62	
MUL_0161	-	hypothetical protein	COG2076P	63	+
MUL_4655	*cysE_1*	serine acetyltransferase CysE_1	COG1278K	63	
MUL_2839	-	hypothetical protein	-	64	
MUL_2263	-	hypothetical protein	COG0793M	64	
MUL_1479	*trxB1*	thioredoxin TrxB1	COG0526OC	66	
MUL_4386	-	hypothetical protein	COG0620E,COG1309K	67	
MUL_5055	*esxF*	ESAT-6 like protein EsxF	COG4842S	67	
MUL_0327	-	oxidoreductase	COG1028IQR	67	
MUL_3206	-	hypothetical protein	-	67	
MUL_3717	-	hypothetical protein	-	68	
MUL_0010	-	hypothetical protein	-	70	
MUL_3264	*fdxA_1*	ferredoxin FdxA_1	COG1146C	70	
MUL_1435	-	exported protein	COG0704P	70	−
MUL_3277	-	hypothetical protein	-	71	
MUL_3581	-	phage-related integrase	COG0582L	71	
MUL_1216	*tam*	trans-aconitate methyltransferase Tam	COG1522K	72	
MUL_2917	-	hypothetical protein	-	72	
MUL_1771	-	hypothetical protein	COG1670J	72	
MUL_0951	-	hypothetical protein	-	73	
MUL_0889	-	hypothetical protein	-	73	
MUL_0993	-	transcriptional regulatory protein	COG0236IQ	73	
MUL_4846	-	hypothetical protein	-	73	−
MUL_0366	*mcmA2b*	methylmalonyl-CoA mutase alpha subunit, McmA2b	-	73	−
MUL_1003	-	hypothetical protein	-	74	
MUL_4682	-	hypothetical protein	COG3391S	74	
MUL_4870	-	short chain dehydrogenase	COG1028IQR	74	
MUL_0457	-	hypothetical protein	COG2351R	74	
MUL_5109	-	hypothetical protein	-	75	
MUL_0761	-	hypothetical protein	-	76	
MUL_1274	*lppN*	lipoprotein LppN	-	76	
MUL_2490	-	hypothetical protein	-	76	
MUL_0820	-	methyltransferase	COG0500QR,COG2226H	76	
MUL_2645	-	AsnC family transcriptional regulator	COG0526OC	76	−
MUL_3440	-	hypothetical protein	COG2185I	77	
MUL_3194	-	hypothetical protein	COG2261S	77	
MUL_0424	-	hypothetical protein	-	77	
MUL_5032	*mpt64*	immunogenic protein Mpt64	COG0425O	77	
MUL_4365	-	hypothetical protein	COG0393S	77	
MUL_4394	-	hypothetical protein	COG0526OC	78	
MUL_3305	*ribD*	hypothetical protein	COG1985H	78	
MUL_3524	-	diphosphomevalonate decarboxylase	COG3407I	78	
MUL_0217	*lpqV*	lipoprotein LpqV	-	78	
MUL_0241	*bioF2_1*	8-amino-7-oxononanoate synthase BioF2_1	COG0156H	79	
MUL_5058	-	hypothetical protein	-	79	
MUL_4336	-	PE family protein	-	79	
MUL_4899	-	hypothetical protein	-	80	−
MUL_4670	-	hypothetical protein	-	82	−
MUL_2937	-	ArsR-type repressor	COG1846K	82	−
MUL_0017	*trpG*	para-aminobenzoate synthase component II	COG1695K	86	−
MUL_5067	*trxC*	thioredoxin TrxC	COG1522K	89	−
MUL_2060	-	hypothetical protein	-	92	−
MUL_0670	*rimL*	acetyltransferase, RimL	COG0664T	92	−
MUL_4330	-	hypothetical protein	-	96	−
MUL_0430	-	hypothetical protein	COG2608P	99	−
MUL_1434	-	hypothetical protein	-	99	−
MUL_0058	-	transcriptional regulatory protein	COG1670J	101	−
MUL_1897	-	ABC transporter ATP-binding protein	-	105	−
MUL_5123	-	hypothetical protein	COG3576R	105	−
MUL_4776	-	hypothetical protein	COG1309K	107	−
MUL_2966	-	hypothetical protein	-	110	−
MUL_5035	-	hypothetical protein	COG0792L	111	−
MUL_0441	*phoY2*	phosphate-transport system regulatory protein, PhoY2	-	112	−
MUL_1835	*secG*	preprotein translocase subunit SecG	-	117	−
MUL_0825	-	hypothetical protein	COG1359S	122	−
MUL_4918	*mce6B*	MCE-family protein Mce6B	COG1463Q	129	−
MUL_2369	-	hypothetical protein	-	132	−
MUL_0065	-	hypothetical protein	COG2353S	136	−
MUL_0035	-	DNA-binding protein	COG1045E	136	−
MUL_3556	-	integral membrane protein	COG0454KR	140	−
MUL_0015	-	putative septation inhibitor protein	COG4842S	141	−
MUL_2243	*esxR*	ESAT-6 family protein	-	146	−
MUL_4627	-	hypothetical protein	-	147	−
MUL_2232	-	molecular chaperone (small heat shock protein)	COG3585H	150	−
MUL_3337	-	hypothetical protein	-	152	−
MUL_3714	*rplU*	50S ribosomal protein L21	COG0261J	156	−
MUL_3030	*ureB*	urease beta subunit UreB	COG0832E	156	−
MUL_4051	-	hypothetical protein	-	165	−
MUL_4256	*whiB4*	transcriptional regulatory protein Whib-like WhiB4	-	183	−

1 “+” represents potential diversifying selection indicated by *p_N_−p_S_* values higher than mean + 3× standard deviation; “−” represents potential negative selection indicated by *p_N_−p_S_* values lower than mean−3× standard deviation.

There has been growing evidence suggesting that some of the ESAT-6 family proteins are involved in the interplay between host and pathogen via either antigenic variation or antigenic drift [38]. ESAT-6 protein encoding genes (*esxA esxB*) are deleted in *M. ulcerans* strains of the classical lineage [4],[39], which could contribute to antigen variation and enable the pathogen to escape the immune defense of the host. The Agy99 genome contains 12 genes encoding ESAT-6 like proteins [4]. This may represent a genomic basis for antigenic variation. The duplicated genes might encode antigenically different proteins with the same function. The differential expression of individual genes could enable them to substitute for each other functionally but escape from host immune recognition. The high number of SNPs and significantly high/low *p_N_*−*p_S_* values we observed here on ESAT-6 family protein genes such as *exsE*, *esxF*, *esxR*, and another secreted antigenic protein gene *mpt64*, may be the result of selective pressure imposed by the immune system of the host. Mutations that lead to replacement of amino acids within the immunodominant epitopes have been proposed as a mechanism producing antigenic drift [40]. However, it remains to be investigated whether the esx genes are expressed, if their expression is controlled and coordinated, and if the mutations identified here lead to antigenic drift.

We further examined whether certain functional classes of genes were under positive selection by comparing *p_N_*−*p_S_* values according to the Clusters of Orthologous Group (COG) classification. We found no significant overrepresentation of any functional class in gene group with positive *p_N_*−*p_S_* values (under diversifying selection), except the “Function unknown” class (p value <0.05). Five functional classes showed overrepresentation of genes with negative *p_N_*−*p_S_* values, indicating possible selections against amino acid changes, including functional classes J (translation, ribosomal structure and biogenesis), O (posttranslational modification, protein turnover, chaperones), F (nucleotide transport and metabolism), H (coenzyme transport and metabolism) and V (defense mechanisms).

### Phylogeny and estimation of the divergence time of *M. ulcerans* strains

**Figure 4:**
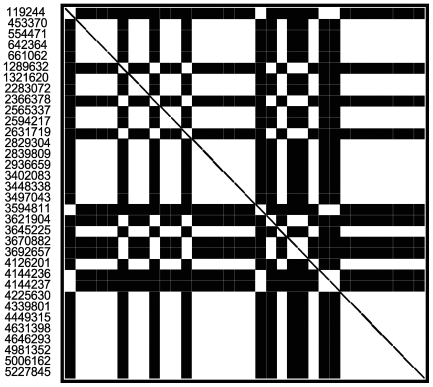
Compatibility matrix of parsimony informative SNPs. The genome positions are numbered to the left of the matrix. Black squares indicate incompatible sites, where nucleotide changes are inferred to have occurred multiple times either due to recombination or repeated mutation. White squares represent compatible sites, at which all nucleotide changes can be inferred to have occurred only once in a phylogeny.

To evaluate the phylogenetic relatedness of the *M. ulcerans* strains sequenced in this study, we first analyzed the 34 PI SNP sites using the compatibility matrix program [41] to detect the effects of recombination on sequence divergence among the genes harboring these SNPs (Figure 4). In the square matrix, each white square corresponds to two compatible nucleotide sites, at which all nucleotide changes can be inferred to have occurred only once in a phylogeny. Black squares represent incompatible sites, where nucleotide changes are inferred to have occurred multiple times either due to recombination or repeated mutation. We found the 34 PI SNPs formed three groups: SNPs shared by the two Ghanaian test strains (at genomic locations of 1289632, 2366378, 2631719, 3621904, 3670882, 3692657), SNPs shared by NM31/04 and Jp8756 (at genomic locations of 119244, 3594811, 4144236, 4144237) and the rest of the SNPs shared by NM20/02 and Jp8756. The SNPs were compatible within each group but not across groups. The overall compatibility score of all 34 PI SNP sites was 0.5294, which measures the extent of the sites consistent with one phylogeny. The neighbor similarity score of the matrix was 0.6506, which was not significantly higher than scores of 1000 random matrices produced by shuffling the order of sites (mean score 0.6168, P = 0.30), suggesting that recombination among these regions has been rare.

**Figure 5:**
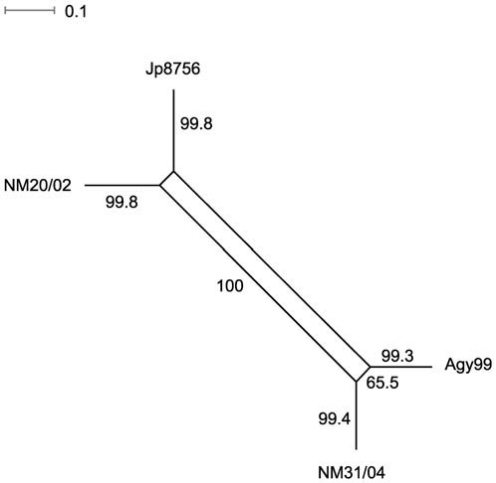
NeighborNet network of the *M. ulcerans* strains based on the parsimony informative SNPs. Bootstrap values shown close to branches are based on 1000 bootstrap replicates.

**Figure 6:**
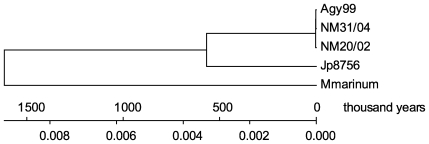
Minimum evolution tree based on 1,032,790 allelic codons of the *M. ulcerans* and *M. marinum* strains. The scale shows the divergence time frame and the number of synonymous substitutions per nucleotide site. The rate of synonymous substitution used for time calibration was 5.8×10^−9^ substitution per site per year.

We then used the split decomposition method to detect possible conflicting phylogenetic signals (Bandelt and Dress 1992). NeighborNet Network analysis of the 34 PI SNP sites revealed two parallel paths indicative of the presence of phylogenetic incompatibilities in the divergence of *M. ulcerans* strains, which could arise from recurrent mutation or recombination in the genomes. However, one parallel path showed much lower bootstrap support value (65.5%) than the other parallel path (100%). The paths with high bootstrap support values (over 99.5%) showed a tree-like network (Figure 5), suggesting that a bifurcating tree is an appropriate model for constructing strain phylogeny. Therefore, we constructed a minimum evolution tree of *M. ulcerans* strains rooted with *M. marinum* M to estimate the divergence time (Figure 6). We first compared the 3,597 SNP harboring *M. ulcerans* genes with the *M. marinum* M genome and identified 3,059 genes that have homologous genes in *M. marinum* M [42]. We then generated concatenated allelic sequences of the 3,059 genes for the three test strains and two reference strains (a combined total of 1,032,790 codons in 3,113 kb of coding sequences per strain), based on which a minimum evolution tree of *M. ulcerans* strains was constructed. Estimated divergence time based on comparison of numbers of synonymous substitutions per nucleotide site (*d_S_*) in the 1,032,790 allelic codons are shown in Table 4. The calculations were based on the estimated rate of synonymous substitution in bacteria of 5.8×10^−9^ to 7.8×10^−9^ substitution per site per year [43]. Our results suggest that Agy99 and *M. marinum* M diverged from a common ancestor about 1.13 to 1.52 million years ago, which confirms the recent divergence of *M. ulcerans* from its *M. marinum* progenitor, and seems more precise than the earlier estimation of between 1.2 and 4.7 million years [3]. The African classical lineage strain Agy99 and the Japanese ancestral lineage strain Jp8756 diverged about 394 to 529 thousand years ago (Table 4). Although the ancestral lineage was found genetically closer to the progenitor *M. marinum* in regions of difference (RD) composition [6], the SNP data suggested the Japanese strain was closer to the Ghanaian strains than to *M. marinum*. The discrepancy between the SNP phylogeny and the RD phylogeny could indicate that regions of *M. ulcerans* genomes harboring these genetic variations have diversified through different mechanisms at variable rates. The discrepancy could also be due to the small number of strains analyzed in our study. Among Ghanaian *M. ulcerans* strains the divergence times is less than 3,000 years. However, there are uncertainties in our phylogenetic estimates because of rate heterogeneity. The two-cluster test in LINTREE showed that all the interior nodes within the Ghanaian clade evolved at a uniform rate, while the Japanese strain evolved at a significantly different rate (CP = 99.96%). The branch-length test further indicated that the Japanese strain evolved significantly slower than average, and that all three Ghanaian strains evolved significantly faster than average. Thus, the divergence time of three Ghanaian strains is likely to be shorter than estimated here.

**Table 4:** Estimation of time of divergence between *M. ulcerans* Agy99 and other *M. ulcerans* and *M. marinum* strains.

Strain	*d_s_* 1	Est. divergence time^2^ (yr)	Est. divergence time^3^ (yr)
NM31/04	0.000017±0.000004	1,466±345	1,090±256
NM20/02	0.00003±0.000006	2,586±517	1,923±385
Jp8756	0.006141±0.000081	529,397±6,983	393,654±5,192
*M. marinum* M	0.017642±0.000138	1,520,862±11,897	1,130,897±8,846

1 mean±standard error

2 Based on the rate of synonymous substitution of 5.8×10^−9^ per site per year

3 Based on the rate of synonymous substitution of 7.8×10^−9^ per site per year

### Genetic diversity among Ghanaian clinical isolates

**Figure 7:**
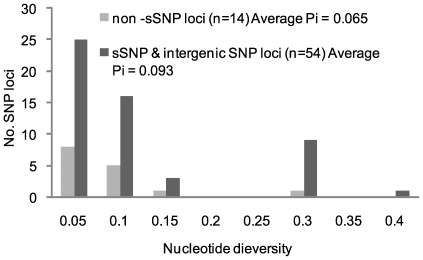
Nucleotide diversity among SNPs identified through genome comparison of three Ghanaian strains, for which complete SNP data have been collected in 54 Ghanaian *M. ulcerans* strains.

**Figure 8:**
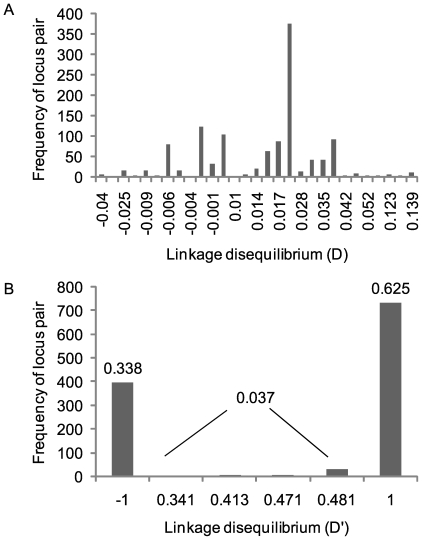
Linkage disequilibrium among study loci. A). The distribution of the linkage disequilibrium coefficient (D) for 1,176 pairwise comparisons of alleles at 54 sSNP and intergenic SNP loci. A total of 751 (64%) of these comparisons are significant by a chi-squared test, and600 (51%) remained significant using a Bonferroni correction for multiple tests. B). The distribution of the standardized coefficient of linkage disequilibrium (D'). Ninety-six percent of the locus pairs are in complete linkage disequilibrium.

We are developing hairpin primer SNP assays [44] based on the 173 SNP loci discovered through pairwise comparisons of the three *M. ulcerans* Ghanaian strains and are analyzing our collection of Ghanaian *M. ulcerans* isolates at these loci. At the current stage it is possible to resolve the nucleotides at 68 SNP loci in 54 Ghanaian strains with 13 distinct SNP haplotypes identified (including the three reference strains). Previously only 3 haplotypes were found in this strain collection by VNTR typing, which was the best resolution achieved [7]. The nucleotide diversity ranged from 0.05 to 0.40 across the 23 sSNP and 31 intergenic SNP loci with an average diversity of 0.09 (Figure 7). Across the 14 non-sSNP, the average nucleotide diversity was 0.06. This level of nucleotide diversity means that two isolates selected at random from this collection will differ at a SNP locus in 6–9% of the cases. The alleles at the sSNP loci were highly nonrandom in their haplotype distribution. This statistical association can be seen in the distribution of the linkage disequilibrium coefficient (D) for 1,176 pairwise comparisons of alleles at 54 sSNP and intergenic SNP loci (Figure 8). A total of 751 (64%) of these comparisons were significant by a chi-squared test, and 600 (51%) were significant using a highly conservative Bonferroni correction for multiple tests [45]. The standardized coefficient of linkage disequilibrium (D′) was strongly U-shaped with 96% of the locus pairs in complete linkage disequilibrium. This observation indicates that there are at most three out of the four possible haplotypes for most locus pairs. It also suggests that recurrent mutation and recombination events have played only a minor role in generating haplotype diversity. Detailed epidemiological analysis of the typing results is out of the scope of this paper and will be summarized elsewhere (manuscript in preparation).

## Discussion

In this study we have sequenced genomes of two Ghanaian and one Japanese *M. ulcerans* disease isolates by using two different massively parallel sequencing platforms. By comparison of genome sequences of these strains with the genome of the Ghanaian reference strain Agy99, we have identified over a hundred SNPs within Ghanaian strains and about 26,000 SNPs in the Japanese strain. The SNPs identified among Ghanaian strains for the first time allow to resolve the population structure and evolutionary relationship of an intra-continental population of *M. ulcerans*. The SNP data confirmed the recent divergence of *M. ulcerans* and *M. marinum* from a common ancestor and refined the estimated time of emergence to about 1.3 million years. We also estimated that the classical *M. ulcerans* lineage (represented by the Ghanaian strains) diverged from the ancestral lineage (represented by the Japanese strain) about 400,000 years ago, which is about the time when the modern human species, *Homo sapiens*, evolved (∼250,000 to 400,000 years). However, we need to point out two limiting factors of our current estimates. First is the limited number of strains analyzed. Future studies should include many more strains from different countries and continents representing branches of the two lineages to improve the phylogenetic resolution and accuracy of the dating. Second is that our dating was based on the estimated rate of synonymous substitution in bacteria of 5.8×10^−9^ to 7.8×10^−9^ substitution per site per year, which was not the most appropriate clock rate for calculating the age of genetically monomorphic pathogens [46]. However, till now studies trying to establish the ideal molecular clock calibrated against a fossil record have not yielded much usable new information [46]. The clock rate we used has been used to date other monomorphic pathogens such as *M. ulcerans* [3], *M. tuberculosis* [43],[47] and *Escherichia coli* [48], and our estimated age of *M. ulcerans* is thus still informative and comparable to these previous estimations.

The SNP analysis suggested that the Japanese strain was closer to the Ghanaian strains than to *M. marinum*, which is contradictory to the previous finding based on RD composition, where the ancestral lineage was found genetically closer to the progenitor *M. marinum* [6]. The discrepancy might suggest that regions of *M. ulcerans* genomes harboring these genetic variations have diversified through different mechanisms at variable rates. However, more strains within the ancestral lineage should be analyzed before firm conclusions can be drawn. Among Ghanaian strains the divergence time was less than 3000 years. Two-cluster and branch-length tests in this study revealed that the Ghanaian strains evolved significantly faster than average, thus the actual divergence time within Ghana could be much shorter than our current estimate. *M. ulcerans* was first isolated in 1948 [49], but large ulcers almost certainly caused by *M. ulcerans* have been already described by Sir Albert Cook in 1897. Since the late 1980s the number of reported cases has increased dramatically in West Africa [50],[51],[52],[53] and in Australia [54]. Increased disease incidence has been primarily attributed to factors such as environmental changes, increased exposure of the affected populations, and improved surveillance. VNTR typing results gave indications for the emergence and spreading of new genetic variants of *M. ulcerans* within Ghana [7]. More in-depth phylogenetic and functional analyses are needed to test if mechanisms such as virulence evolution and host adaptation of *M. ulcerans* play a role in the increasing incidence of BU.

Genome comparison of *M. ulcerans* Agy99 and *M. marinum* M revealed that *M. ulcerans* underwent reductive evolution with genomic signatures such as proliferation of ISE, accumulation of pseudogenes, chromosomal rearrangements, genome downsizing, and acquisition of foreign genes by acquisition of plasmids or bacteriophages [4]. Microarray based comparative genomic hybridization with a worldwide set of *M.* ulcerans isolates identified genomic regions of difference and demonstrated that the two major *M. ulcerans* lineages can be distinguished based on the location and size of genomic deletions [5],[6]. Due to the lack of paired-end sequencing data, we did not carry out systematic analysis on chromosomal deletions and rearrangements. But when we tried to close sequencing gaps using the Agy99 chromosome as the reference, there were more successful gap closure reactions in Ghanaian strains than in the Japanese strain. Failed gap closure attempts in Ghanaian strains were mostly around IS elements, while this was not the case for the Japanese strain (data not shown). These results suggest a high frequency of large chromosomal rearrangement events in the Japanese strain compared to the African classical lineage strains. In the two Ghanaian strain, NM31/04 and NM20/02, the plasmid was fully covered by sequence reads and almost identical to pMUM001 in Agy99, while the plasmid in the Japanese strain was only partially covered with low depth. Altogether these observations suggest a more stabilized genome and a less important role of reductive evolution within *M. ulcerans* Ghanaian strains.

SNP analysis revealed no further pseudogene formation within the Ghanaian strains, and the Ghanaian strains were found to evolve significantly faster than average. While the majority of coding region SNPs found in Jp8756 was synonymous, the majority found in the two Ghanaian strains was non-synonymous. Further functional analysis of genes containing these non-sSNPs would help to elucidate if they could lead to “pathoadaptive” niche expansion, or provide a selective advantage in both sporadic infection and epidemic spread, which have been found in other bacterial pathogens [55],[56] and suggested for *M. ulcerans* [39],[57].

The low genetic diversity and high linkage disequilibrium within Ghanaian isolates supports the hypothesis that the *M. ulcerans* population spread over the African continent has gone through a severe bottleneck during adaptation to a possibly host-specific environment and has not yet accumulated much sequence diversity [15]. SNP typing of Ghanaian isolates was consistent with VNTR typing [7], but allowed to further differentiate between isolates coming from the same BU endemic focus. Closely related, but distinct clonal complexes including strains with minor variation seem to dominate in different BU endemic areas. Diversity of these local clonal complexes is indicative for ongoing microevolution.

Although there has been impressive recent progress in studying the transmission of BU, the precise environmental reservoirs and mode(s) of transmission are not fully understood [58],[59]. High-throughput genotyping platforms, such as Hairpin primer real time PCR assays [44], BeadArray [60] and OpenArray [61] will make genome wide SNP typing a highly discriminatory and cost-effective tool to study *M. ulcerans* evolution and epidemiology at a population scale. More in-depth phylogenetic and phenotypic analyses of a large number of disease isolates and environmental strains (once becoming available) is expected to shed more light into transmission and virulence evolution of *M. ulcerans* after its divergence from *M. marinum*. The study will also help to identify SNPs associated with host specificity and geographical origins. However, the current set of SNP markers were obtained by comparing genomes of three very closely related Ghanaian strains and one very distant Japanese strain. Future genome sequencing of more representative strains from diverse locations around the world will be necessary to identify additional SNP markers to delineate the origin and spread of *M. ulcerans* at both local and global level.

## Materials and Methods

### Bacterial strains and genomic DNA isolation

The three *M. ulcerans* strains sequenced with various sequencing platforms are listed in Table 1. Strain Jp8756 (ATCC 33728) from Japan was provided by Francoise Portaels (Institute of Tropical Medicine, Antwerp, Belgium). Isolation and characterization of strains NM31/04 and NM20/02 from Ghana has been described elsewhere [7],[8],[62],[63]. We used the complete Agy99 genome sequence (chromosome, NC_008611, and the plasmid pMUM001, NC_005916) as the reference sequences. Genomic DNA was isolated as described [64].

### Pyrosequencing of *M. ulcerans* NM20/02

We sequenced the genome of *M. ulcerans* NM20/02 using the Roche 454 Life Sciences Genome Sequencer FLX following the manufacturer's instructions (Roche 454 Life Science, Branford, CT, USA). The shotgun library was prepared with 5 µg genomic DNA using the “Standard DNA Library Preparation Kit” (04852265001, Roche). Nebulized, purified, and adaptors attached single strand DNA fragments were clonally amplified using the “Emulsion PCR Kit I” (04852290001, Roche). Sequencing on the GS FLX was performed using the “Standard LR70 Sequencing Kit” (04932315001, Roche). Images were processed using the “Genome Sequencer FLX Data Processing Pipeline 1.1.02.15”. A total of 424,494 reads with average length of 213 bases were obtained.

We mapped 454 reads to the reference sequence using gsMapper (v1.1.02.15, Roche) to make SNP calls. To identify high confidence SNPs, we filtered substitutions reported by gsMapper using two rules: (1) supported by at least three non-duplicated reads, with at least one aligned in the forward direction and at least one aligned in the reverse direction. (2) Requirement for at least five conserved bases on both sides of the SNPs. We randomly selected 37 SNPs distributed across the genome for PCR and capillary sequencing. 29 SNPs were validated, three were miscalled due to sequencing errors in the Agy99 genome, and five failed due to unspecific PCR reactions (Table S3). After manual inspection, we found that each of the five SNPs was supported by all the reads (7 to 16) uniquely mapped to the region, thus was likely to be a true SNP. Unspecific PCR reactions might be due to the presence of genes paralogous to genes harboring or flanking the SNPs.

### Solexa sequencing of *M. ulcerans* NM31/04 and Jp8756

We sequenced the genomes of *M. ulcerans* NM31/04 and Jp8756 with the Illumina Genome Analyzer according to the manufacturer's instructions (Illumina, San Diego, CA, USA). The DNA Colony/Cluster Template Library was prepared using the Illumina “Genomic DNA Sample Prep Kit” (Illumina). Briefly, 5 µg of genomic DNA was broken into fragments of approximately 100 bp by nebulization. After end repairing and adaptor ligation, the samples were gel-purified to recover fragments of 150–250 bp, which were PCR amplified for 15 cycles. For quality control, an aliquot of the library was cloned into a TOPO plasmid. Six clones from each bacterial strain were picked and subjected to capillary sequencing. The DNA Colony/Cluster Template Library was then used for flow-cell preparation using the “Standard Cluster Generation Kit” (Illumina). Sequencing on the Illumina Genome Analyzer was performed using “Genomic DNA sequencing primer V2” for 36 cycles. At the end of the run, images were processed using the “Solexa Data Analysis Pipeline 0.2.2.6”. A total of 2.538 and 2.651 million reads of 35 bases in length were obtained for NM31/04 and Jp8756, respectively.

We mapped Solexa reads to the reference sequences using MAQ v0.6.3, which is particularly developed for building mapping assemblies from Illumina Solexa reads. For each read, un-gapped alignment against the reference was performed; all hits with up to 2 mismatches in the first 24 bp were found. Each read was placed to the position where the sum of quality values of the mismatched nucleotides is minimum [33]. For detection of high confidence SNPs, we first filtered the SNP calls reported by MAQ based on two rules: (1) supported by more than three non-duplicated reads; (2) covered by at least one read with a mapping quality higher than 40. We then randomly selected 84 SNPs called in NM31/04 or Jp8756 for PCR and capillary sequencing to determine the cutoff value of the consensus quality. We found that the consensus quality equal or higher than 40 excluded most false positive SNP calls and that the false positive rate was about 1% (Figure S3).

### NimbleGen comparative genome sequencing (CGS) of selective regions in *M. ulcerans* Jp8756

Before next-generation sequencing became widely available, NimbleGen CGS was the cost effective tool for the comparative analysis of microbial genomes to identify SNPs, insertions, or deletions with high speed and accuracy [65]. We first used a mutation mapping array tiling the reference genome to locate potential mutation harboring sites. Then a high density re-sequencing array tiling the putative sites was produced to identify the mutations [66]. The method has been applied successfully to survey the entire or partial genomes of several bacteria [48],[67],[68], including *M. tuberculosis* [69].

For CGS analysis of *M. ulcerans* Jp8756, we selected 1,265 of the 4,160 protein coding genes across the chromosome of Agy99 (1,210,734 bp out of 5,805,761 bp, 20%) and 51 of the 81 protein coding genes on the plasmid pMUM001 (135,612 bp out of 174,155 bp, 78%) (Table S2). These genes include drug resistance genes, known antigens, genes with housekeeping roles, and genes of hypothetical proteins. To avoid cross hybridization and ambiguous SNP calls, we excluded protein coding genes with paralogs in the *M. ulcerans* genome. Highly conserved genes between *M. ulcerans* and its ancestor *M. marinum* were also excluded. Mutation mapping and re-sequencing probes were designed for selected gene using the ArrayScribe software, synthesized using the Maskless Array Synthesis (MAS) technology [70],[71], and printed in a random layout. Genomic DNA samples from Jp8756 and the reference Agy99 were hybridized to the arrays separately following the NimbleGen protocol. Data were analyzed using NimbleScan software (NimbleGen). A list of 1,619 identified SNPs is provided in Table S2.

### SNP validation

We used Sanger sequencing of PCR products to validate a selected subset of SNPs. Primer sequences used for PCR and sequencing are provided in Table S3. PCR products were purified using the NucleoSpin Extract II Kit (Clontech Laboratories, Mountain View, CA).

### SNP typing

We are developing hairpin primer (HP) assays [44] for SNPs discovered through pairwise comparisons of the three *M. ulcerans* Ghanaian strains (Agy99, NM20/02, and NM31/04) and analyzing our collection of clinical isolates from Ghana [7]. All HP assays were tested on the three reference strains to confirm the presence of each allele and to verify the performance of the SNP assays. Assays on the clinical DNA samples were considered reliable only if the cycle thresholds generated in the paired wells differed by three or more cycles. At the reported stage, it was possible to assign alleles to 68 SNP loci in 54 Ghanaian isolates (including the reference strains) using this approach.

### Phylogenetic analysis

To identify putative regions of recombination or gene conversion, we used the Reticulate program and constructed a compatibility matrix [41]. We also used the SplitsTree program to detect conflicting phylogenetic information and determine if a bifurcating tree is an appropriate model to construct strain phylogeny [72]. A minimum evolution tree rooted with *M. marinum* was constructed by using the MEGA [73] software based on the numbers of synonymous substitutions per nucleotide site in concatenated SNP harboring protein coding genes in all strains. The numbers of synonymous substitutions per synonymous site were calculated from the concatenated nucleotide sequences using the modified Nei-Gojobori Jukes Cantor method. The Complete deletion method was used for handling alignment gaps. Because rate heterogeneity may have contributed to uncertainty in phylogeny estimates, we applied the two-cluster and branch-length tests in LINTREE [74] to identify significant rate heterogeneity in the phylogeny. The two cluster test was used to test the molecular clock hypothesis for the two lineages above each interior node of a tree and the branch-length test was used to examine the deviation of each root-to-tip branch length relative to the average length [74]. To generate the concatenated sequences, we first determined homologous genes in the *M. marinum* M genome [42] for each of the 3,597 protein coding gene harboring SNPs by standalone BLAST search [75]. Using the threshold of 90% nucleotide sequence identity over a minimum alignment length of 90% of both query and hit genes, 3,059 homologous genes were identified. Protein sequences of the homologous genes were aligned using the CLUSTALW program [76]. Homologous genes were then aligned using the EMBOSS [77] Tranalign program so that the corresponding amino acid sequence alignment was imposed on the DNA sequence alignment. Allelic genes in *M. ulcerans* strains and homologous genes in *M. marinum* M with alignment gaps were then concatenated.

### Accession numbers

The sequencing reads of the NM20/02, NM31/04 and Jp8756 genomes have been deposited in the NCBI Short Read Archive database under the accession number SRA008258.

## Supporting Information

Figure S1 Coverage of the plasmid pMUM001 by NM20/02 GS FLX reads (A, B), NM31/04 Solexa reads (C, D) and Jp8756 Solexa reads (E, F). X axes represent genomic region of pMUM001 (1 to 174,155 ). The Y axes show the coverage depth. In A, C, and E, all mapped reads were recorded and reads mapped to multiple locations were counted multiple times, while only uniquely mapped reads were counted in B, D, and F.
(0.30 MB PDF)

Figure S2 Absence of pMUM001 plasmid genes in Jp8756 revealed by NimbleGen microarrays. Most hybridization signal intensities from Jp8756 (in Green) were close to zero in both mutation mapping and re-sequencing of selected pMUM001 plasmid genes (A and B), while hybridization signal intensities from Agy99 (in Blue) were properly detected. Hybridization signal intensities from both strains were properly detected in mutation mapping and re-sequencing of selected chromosomal genes (C and D). X axes represent genomic location of oligo probes (1 to 174,155 in A and B, 1 to 5,631,606 in C and D). Y axes show h hybridization signal intensities.
(2.45 MB EPS)

Figure S3 Empirically determined MAQ consensus quality scores improved the accuracy of SNP calls in *M. ulcerans* Solexa data. Sanger sequencing of potential SNP harboring regions in Jp8756 and NM31/04 revealed that MAQ consensus quality scores equal or higher than 40 excluded most false positive SNP calls.
(0.71 MB EPS)

Table S1 SNPs detected among four *M. ulcerans* genomes. Coordinates correspond to Agy99 finished genome sequence (NCBI:NC_008611 for Agy99 chromosome and NCBI:NC_005916 for Agy99 pMUM001).
(7.21 MB XLS)

Table S2 SNPs identified by NimbleGen re-sequencing array in selected chromosomal and plasmid genes. SNPs confirmed by Solexa Genome Analyzer are indicated in the last column.
(0.52 MB XLS)

Table S3 Primers used for PCR and sequencing of SNP harboring regions.
(0.05 MB XLS)
