# Supplementary figures and images for: Genomic Diversity and Evolution of Mycobacterium ulcerans Revealed by Next-Generation Sequencing
Source: PLoS Pathog. 2009 Sep 11;5(9):e1000580. doi: 10.1371/journal.ppat.1000580 (PMC2736377; doi:10.1371/journal.ppat.1000580)

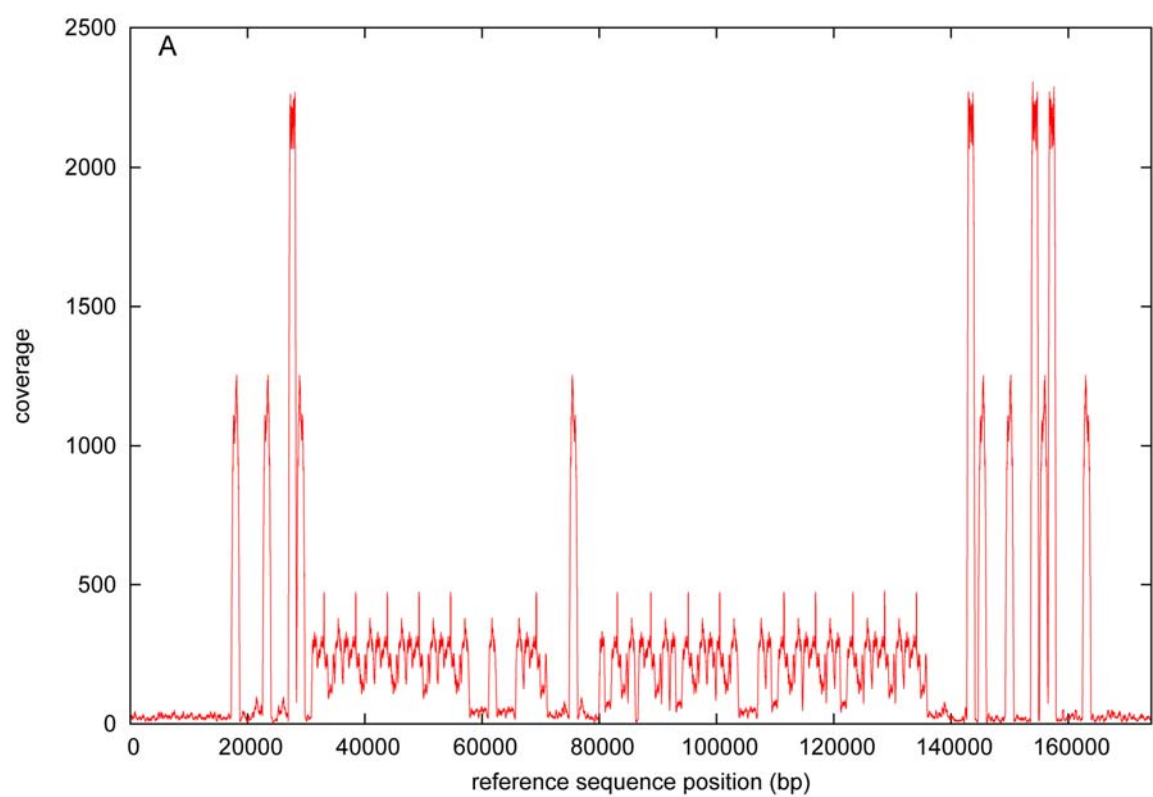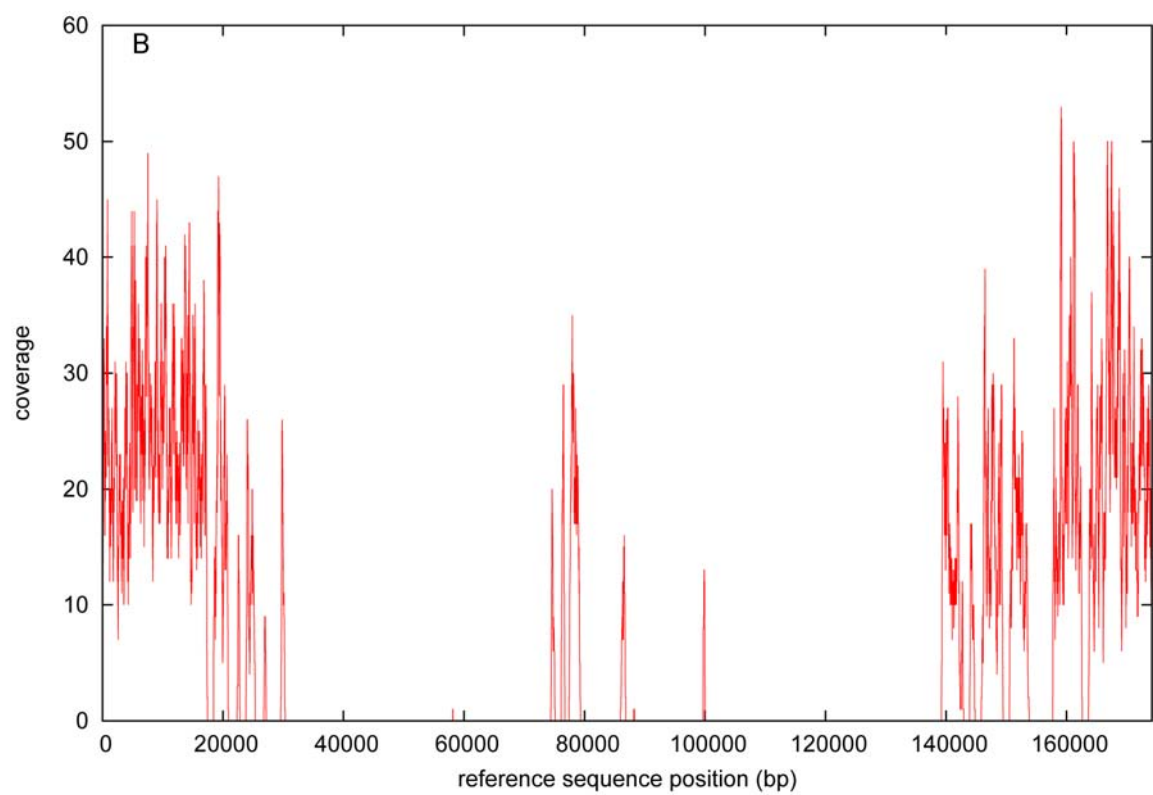

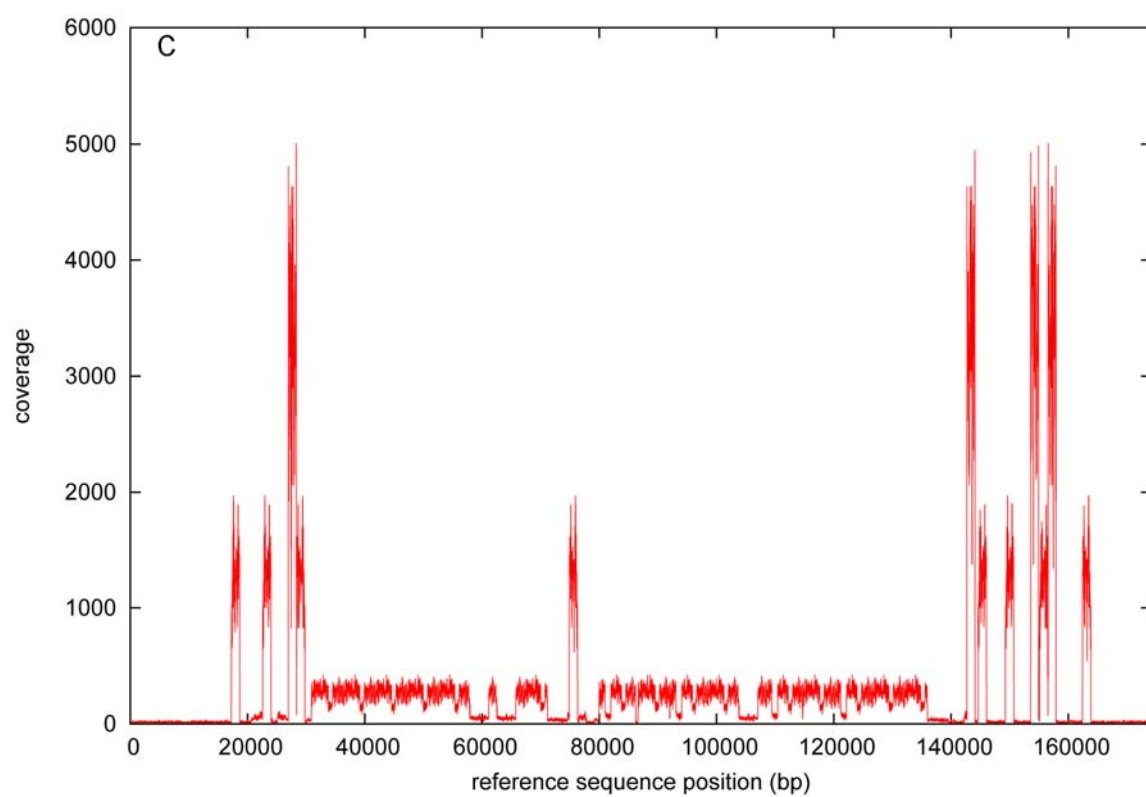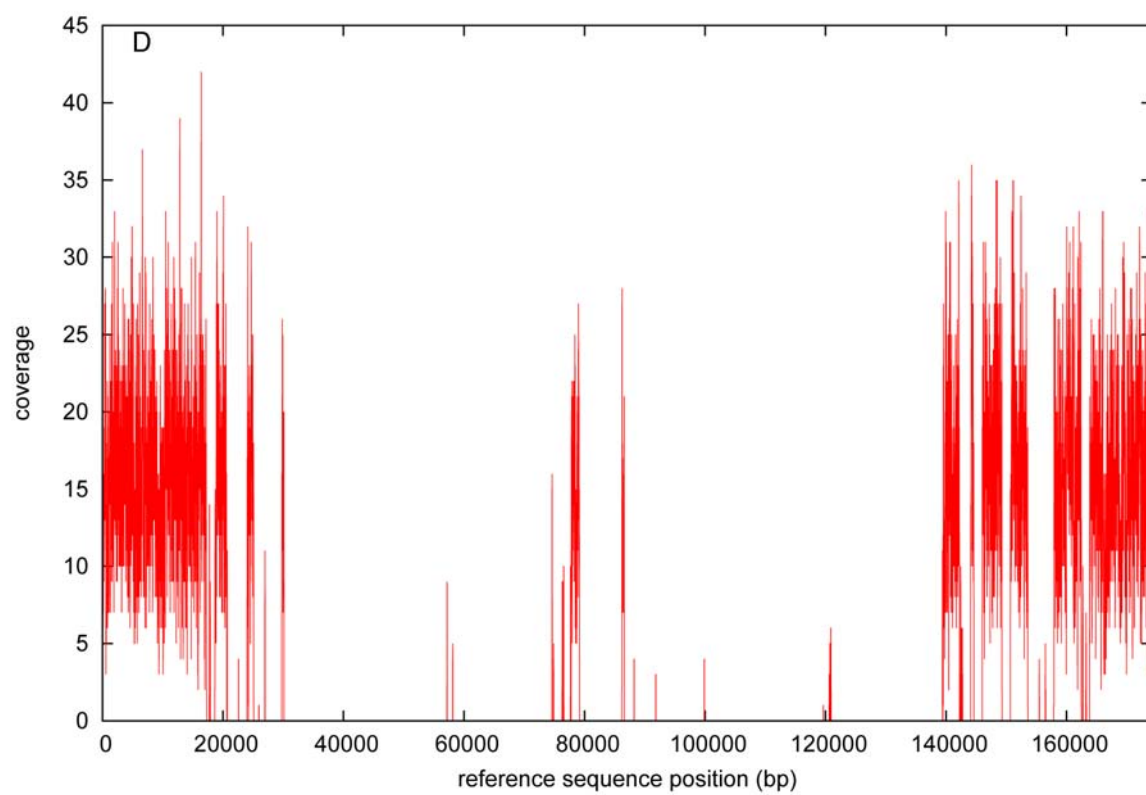

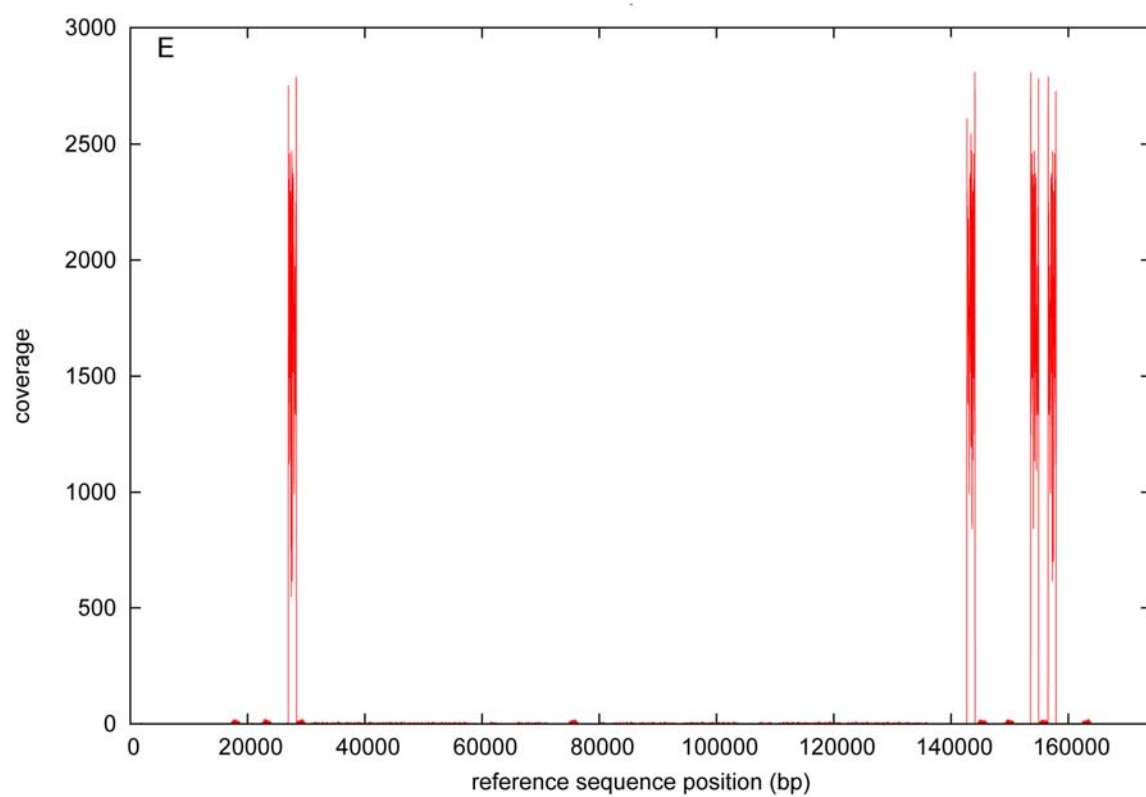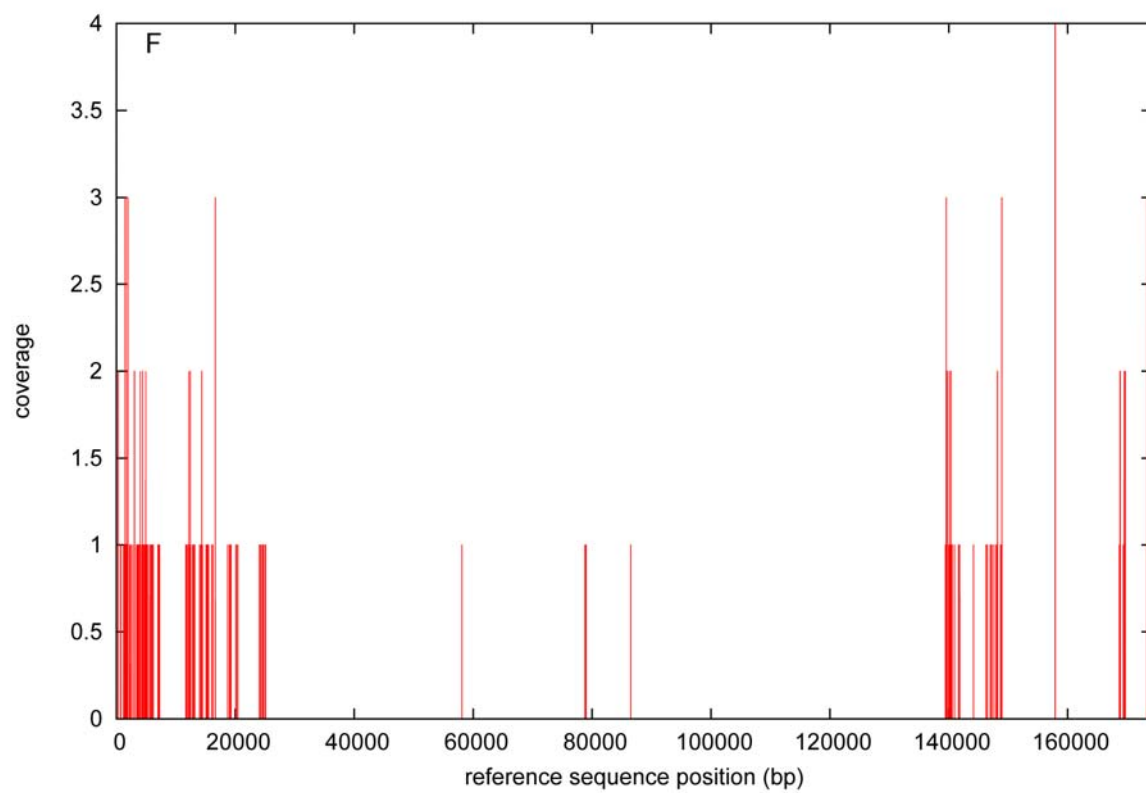

Supplement: Figure S1 — Coverage of the plasmid pMUM001 by NM20/02 GS FLX reads (A, B), NM31/04 Solexa reads (C, D) and Jp8756 Solexa reads (E, F). X axes represent genomic region of pMUM001 (1 to 174,155 ). The Y axes show the coverage depth. In A, C, and E, all mapped reads were recorded and reads mapped to multiple locations were counted multiple times, while only uniquely mapped reads were counted in B, D, and F. (0.30 MB PDF) [file ppat.1000580.s001.pdf]
